# Artificial intelligence in medical and biological research: promise and perils of ChatGPT and DeepSeek in advancing healthcare

**DOI:** 10.55730/1300-0152.2765

**Published:** 2025-10-11

**Authors:** Mahmut Enes KAYAALP, Onur GÜLTEKİN, Serhat AKÇAALAN, Hamit Çağlayan KAHRAMAN, Hüseyin Nevzat TOPÇU, Gülşah KAVRUL KAYAALP

**Affiliations:** 1 Department of Orthopaedics and Traumatology, University of Health Sciences, İstanbul Fatih Sultan Mehmet Training and Research Hospital, İstanbul, Turkiye; 2 Department of Orthopaedics and Traumatology, Brandenburg Medical School Theodor Fontane, University Hospital Brandenburg/Havel, Brandenburg/Havel, Germany; 3 Faculty of Health Sciences Brandenburg, Brandenburg Medical School Theodor Fontane, Brandenburg/Havel, Germany; 4 Department of Orthopaedics and Traumatology, Ankara City Hospital, Ankara, Turkiye; 5 Department of Orthopaedics and Traumatology, Istanbul Kartal Dr. Lütfi Kırdar City Hospital, İstanbul, Turkiye; 6 Department of Paediatric Rheumatology, Pendik Research and Training Hospital, Marmara University, İstanbul, Turkiye

**Keywords:** Artificial intelligence, large language models, ChatGPT, DeepSeek, clinical decision support, medical education

## Abstract

**Background/aim:**

Artificial intelligence (AI), particularly large language models (LLMs) such as ChatGPT and DeepSeek, is being increasingly applied in clinical care, research, and education. The aim of this review is to examine how these tools may transform the conduct of medical and biological research and to define their limitations.

**Materials and methods:**

A narrative synthesis of the literature was performed, encompassing studies published between 2020 and 2025. Peer-reviewed journals, systematic reviews, and high-impact original research articles were included to ensure an evidence-based overview. The principle applications, validation metrics, and clinical implications across orthopedics, oncology, cardiology, internal medicine, and the biological sciences were analyzed.

**Results:**

LLMs demonstrate strong potential in supporting physicians during clinical decision-making, enhancing patient education, and assisting researchers in their work. They are valuable for language-related tasks and for generating structured, clear, and comprehensible content. However, concerns persist regarding data privacy, algorithmic bias, factual accuracy, and excessive dependence on data-driven outputs. Responsible implementation requires safeguards such as human oversight, model transparency, and domain-specific training.

**Conclusion:**

AI tools such as ChatGPT, DeepSeek, and similar models are transforming the way healthcare is delivered and studied. Their current capabilities appear highly promising. However, clinicians, technical experts, and policymakers must collaborate to ensure the safe, equitable, effective, and ethical integration of these technologies into real-world healthcare workflows.

## 1. Introduction

A paradigm shift in clinical care, research, and education has been driven by the integration of artificial intelligence (AI) into the biological and medical sciences. In particular, the emergence of large language models (LLMs), such as ChatGPT, has extended the reach of AI beyond traditional data-processing tasks. These models are utilized to support clinical decision-making, facilitate scientific writing, personalize medical education, and streamline research workflows (Kaarre et al., 2023; Kayaalp et al., 2024; Ayık et al., 2025; Gültekin et al., 2025)

LLMs, powered by transformer-based architectures, are trained on large-scale text corpora and optimized to generate contextually relevant, human-like outputs. ChatGPT, in particular, has demonstrated potential in generating readable scientific abstracts, assisting with grant proposal preparation, and guiding literature searches, especially for researchers facing language barriers (Atik, 2024; Kayaalp et al., 2024). In clinical settings, it has been applied to enhance patient education, develop rehabilitation protocols, and augment diagnostic reasoning through symptom checking and differential diagnosis generation (Kaarre et al., 2023; Cascella et al., 2023; Yapar et al., 2024). In medical education, LLMs offer adaptive learning opportunities, clarifying complex anatomical or pathological content in a user-specific format (Zarei et al., 2024).

Recent evaluations have demonstrated ChatGPT’s competence in standardized medical examinations such as the USMLE (Kung et al., 2023), suggesting that these models can process and generate responses consistent with advanced medical reasoning.

However, concerns persist. These include the phenomenon of hallucination, in which the model generates plausible yet incorrect or misleading information, and limitations in handling nuanced, domain-specific clinical scenarios (Lund et al., 2023; Kayaalp et al., 2025). Additionally, ethical and regulatory frameworks have yet to keep pace with the rapid integration of AI into healthcare, raising questions regarding authorship, data ownership, bias, and accountability (Jeyaraman et al., 2023; Atik et al., 2024).

Importantly, although ChatGPT and other general-purpose LLMs demonstrate considerable promise, they are not specifically fine-tuned for medical or biological applications. Their use should be regarded as supplementary rather than authoritative. As emphasized in a previous editorial (Atik et al., 2024), the primary function of AI should be to enhance the decision-making process and support professionals rather than replace them. Furthermore, their role in research dissemination—such as assisting nonnative English speakers in scientific publishing—highlights both their practical value and the ethical challenges that must be addressed to safeguard scientific integrity (Kayaalp et al., 2025).

This review aims to explore the promises and pitfalls of ChatGPT in medical and biological research. The following sections will examine the historical development of AI in healthcare, explain the architecture of LLMs, and critically assess real-world applications in clinical research, patient care, and scientific publishing. Additionally, emerging multimodal models such as DeepSeek will be explored, followed by a discussion of limitations related to trust, transparency, and bias, and evidence-based recommendations for the responsible integration of AI into healthcare systems will be provided.

## 2. Historical background of artificial intelligence in medical and biological research

The integration of artificial intelligence (AI) into medical and biological research has followed an evolutionary path. This trajectory extends from the deterministic logic of early expert systems to the probabilistic reasoning of modern deep learning models and large language architectures. Each developmental milestone reflects a deeper convergence between data science and the clinical as well as biological domains.

It is estimated that the doubling time of medical knowledge was 50 years in 1950, 7 years in 1980, and 3.5 years in 2010. In 2020, it was projected to be approximately 73 days^1^. While newer estimates are yet to be validated, the trend underscores the increasing burden on researchers to keep pace with scientific literature. The earliest applications of AI in healthcare during the 1970s focused on rule-based expert systems such as MYCIN (Shortliffe, 1976), which encoded human expertise into decision trees to identify infections and recommend appropriate treatments. INTERNIST-I was another pioneering system in general internal medicine (Miller et al., 1982). However, these systems were constrained by their inability to accommodate the uncertainty and variability inherent in clinical data, which significantly limited their practical applicability (Kumar et al., 2022) .

This limitation prompted the emergence of machine learning (ML) in the 1990s and early 2000s, in which statistical methods replaced hand-coded rules. In medical domains, ML enabled predictive modeling across diagnostics, risk stratification, and surgical outcomes. For instance, the application of ML algorithms to large-scale surgical databases such as the Danish Hip Arthroscopy Registry (DHAR) enabled the prediction of revision risk following hip procedures. Martin et al. developed models such as Cox elastic net and super learner ensemble regressors, demonstrating that even with advanced algorithms, performance remained moderate and highly dependent on data quality and standardization. This underscored the necessity of structured, high-fidelity clinical registries for AI tools to provide reliable clinical guidance (Martin et al., 2023).

Deep learning (DL), particularly after the 2012 ImageNet breakthrough, enabled algorithms to learn high-dimensional representations directly from raw data without manual feature extraction. In biomedical contexts, DL revolutionized imaging diagnostics, histopathology, and genomics, outperforming traditional models in complex classification tasks. Nonetheless, despite improvements in accuracy, the opacity of these “black-box” models continued to pose challenges for clinician trust and regulatory acceptance.

A major leap occurred with the advent of natural language processing (NLP) tools and large language models (LLMs) such as BERT and GPT. These models were trained on vast biomedical corpora, equipping them to understand, synthesize, and generate human-like medical text.

Moreover, the role of generative AI in orthopedic research has expanded beyond educational purposes. Ollivier et al. highlighted its application in systematic reviews, manuscript drafting, and patient communication, emphasizing the productivity gains it offers to researchers who are not fluent in English (Ollivier et al., 2023). Similarly, it has been proposed that the responsible deployment of ChatGPT for manuscript generation could reduce language barriers in scientific publishing and help level the playing field for nonnative English speakers (Kayaalp et al., 2024).

In summary, the historical trajectory of AI in medical and biological sciences—anchored in early expert systems, accelerated by machine and deep learning, and redefined by LLMs—reflects both technological ingenuity and the continuing need for clinically grounded applications. The expanding body of referenced literature exemplifies the multifaceted evolution of AI as it transitions from theoretical promise to clinical utility, as will be described in detail below.

## 3. Large language models such as ChatGPT and DeepSeek: Foundations and mechanisms

The foundation of LLMs, including ChatGPT and DeepSeek, lies in their capacity to process vast textual data through advanced neural architectures that emulate aspects of human language understanding. Their development has opened new frontiers in medical and biological research by enabling automated text generation, summarization, and patient education at an unprecedented scale and level of fluency.

At the core of ChatGPT and similar models lies the transformer architecture, which revolutionized natural language processing by replacing sequential modeling with self-attention mechanisms, enabling the model to assess the relevance of each word in a sentence simultaneously. This innovation substantially improved long-range dependency management and computational efficiency—two critical attributes for analyzing biomedical texts that frequently involve complex terminologies and nested semantic structures (Cheng et al., 2023).

Training an LLM involves two stages: pretraining and fine-tuning. During the pretraining phase, models are exposed to massive and diverse text datasets to learn grammar, syntax, semantics, and general world knowledge. Fine-tuning adapts the model for specific tasks, such as medical summarization or clinical question answering, by retraining it on curated, domain-specific datasets. As noted by Cascella et al. (2023), this dual-phase training underpins the versatility of LLMs in clinical applications ranging from literature review automation to diagnostic decision support (Cascella et al., 2023).

A critical component of this process is tokenization, which decomposes text into manageable units—words, subwords, or characters—allowing the model to map semantic meaning efficiency. This step is particularly crucial in medical contexts where acronyms, rare terms, and nonstandard phrasing are common. The attention mechanism further enhances contextual interpretation by assigning adaptive weights to each token according to its semantic relevance, enabling nuanced comprehension of dense, technical narratives.

Despite these architectural advancements, current LLMs have notable limitations. Gültekin et al. (2025) compared ChatGPT-4o and DeepSeek R1 in the context of patient education for anterior cruciate ligament (ACL) surgery. ChatGPT, particularly when using the DeepResearch feature, generated more comprehensive responses, effectively synthesizing complex medical concepts and surgical techniques^2^. However, this improvement came at the expense of reduced readability, particularly for lay audiences, owing to longer and more complex outputs. Conversely, DeepSeek R1 produced more concise and grammatically accessible responses, offering particular advantages for nonnative English speakers and healthcare professionals seeking linguistic precision. These findings highlight that model architecture alone is insufficient. Interface design, prompt engineering, and inference-time optimization also play pivotal roles in determining real-world usability.

Further challenges include limitations in reasoning and factual consistency. Although LLMs excel at generating coherent, probabilistically grounded text, they do not inherently perform logical reasoning. In clinical contexts, particularly in differential diagnosis or treatment planning, this distinction is critical, as confidently stated yet inaccurate outputs may mislead users. Prior research has cautioned against overreliance on LLM-generated content, emphasizing that such models may omit crucial clinical nuances or fail to reference current evidence (Leopold et al., 2023; Kaarre et al., 2023; Kayaalp et al., 2024).

To address these limitations, newer models are being developed using retrieval-augmented generation (RAG) and chain-of-thought prompting, which allow access to validated external knowledge sources and facilitate stepwise reasoning. These innovations aim to enhance factual reliability and transparency, particularly in applications such as clinical education, systematic review drafting, and preliminary diagnostic support in low-resource settings (Huang et al., 2024).

In summary, the foundational architecture of LLMs, comprising transformers, tokenization, attention mechanisms, and fine-tuning, forms a powerful basis for advanced language understanding in medicine. However, their safe and effective implementation in clinical environments requires continuous domain-specific calibration, performance validation, and interface-level refinement.

## 4. Applications of large language models in medical research and practice

### 4.1 Clinical decision-making and application support

Building upon these foundational mechanisms, LLMs such as ChatGPT and DeepSeek are increasingly being integrated into clinical decision support (CDS) systems across diverse healthcare domains. Their ability to process and synthesize unstructured data at scale has created new opportunities for improving diagnostic precision, streamlining surgical planning, and enhancing patient communication (Sagheb et al., 2021).

Equipped with inference-enhancing tools such as DeepResearch and DeepThink, LLMs are capable of simulating clinical reasoning and generating context-aware outputs tailored to specialized clinical queries (Gültekin et al., 2025). These capabilities have been applied not only in direct patient interactions but also in back-end systems designed to structure clinical data for research and registry purposes. For instance, NLP models have demonstrated over 98% accuracy in extracting structured variables—such as prosthesis type, laterality, and intraoperative constraints—from operative notes related to total knee arthroplasty. Similarly, BERT-based models have proven effective in parsing surgical documentation for national registry inclusion, thereby automating processes that were previously manual and error-prone (Sagheb et al., 2021). NLP tools have demonstrated increasing utility in orthopedic research, particularly in extracting variables and identifying patient cohorts from unstructured clinical data such as electronic medical records (Pruneski et al., 2023a).

Complementing LLM-based tools, deep learning models have demonstrated high performance across a range of diagnostic and decision-making tasks in orthopedics. In a retrospective study involving various upper-extremity radiographs, deep learning algorithms accurately detected radius and ulna fractures (Erdaş, 2023). Another study analyzing pelvic radiographs of patients with hip pain identified the potential utility of these models in diagnosing femoroacetabular impingement (Atalar et al., 2023). Similarly, a model developed using over than 11,000 full-leg radiographs demonstrated diagnostic accuracy in lower-extremity alignment comparable to that of an orthopedic surgeon with over a decade of experience (Jo et al., 2023). AI-assisted analysis of more than 8700 long-leg radiographs enabled automated CPAK and functional phenotype classification, revealing significant sex-specific differences in preoperative knee morphology among patients undergoing total knee arthroplasty (Huber et al., 2023).

Syndesmotic instability, a condition involving complex multiplanar deformities, has traditionally presented diagnostic challenges. However, deep learning algorithms applied to weight-bearing CT scans have demonstrated high diagnostic accuracy in identifying this pathology (Borjali et al., 2023). In the domain of ACL reconstruction, where revision surgery remains a notable concern, machine learning models have outperformed conventional statistical methods in reoperation risk prediction, enabling more individualized risk stratification for surgical candidates (Johnson et al., 2023).

In shoulder pathology, machine learning has also demonstrated value. A study evaluating patients with shoulder pain and dysfunction utilized survey and physical examination data, with final diagnoses confirmed through arthroscopy. The resulting model showed high diagnostic accuracy for rotator cuff tears (Li et al., 2023). In the context of osteoarthritis prediction, a recent machine learning study demonstrated high predictive performance in identifying lateral compartment osteoarthritis among patients with symptomatic discoid lateral meniscus. Age emerged as the most important predictive factor overall, while medial compartment osteoarthritis (OA) was a stronger predictor in older patients (Cho et al., 2024). A machine learning model using the random forest algorithm achieved high predictive performance in identifying risk factors for secondary meniscus tears after ACL reconstruction, outperforming traditional logistic regression. Key predictors included early return to sport, delayed surgery, and older age, offering potential value for patient counseling and targeted intervention (Jurgensmeier et al., 2023). Likewise, in meniscal injury evaluation, deep learning algorithms performed well despite a modest dataset. A study analyzing 642 knee MRIs found that deep learning algorithms could effectively localize the meniscus and detect tears with high precision, even when benchmarked against expert orthopedic surgeons (Güngör et al., 2025).

The posterior tibial slope (PTS), a key biomechanical parameter influencing ACL injury and reinjury, is often challenging to measure accurately due to slice-selection variability and methodological differences. Deep learning-based PTS assessment techniques have demonstrated superior reliability and time efficiency compared with manual methods, providing enhanced consistency in clinical decision-making (Yao et al., 2025). In the context of knee osteoarthritis, Kunze et al. (2023) developed a deep learning algorithm capable of performing large-scale automated measurements of limb alignment and length discrepancies from full-limb radiographs. Applied to more than 1000 patients, the model generated over 12,000 measurements within hours and demonstrated excellent agreement with radiologist annotations. Their population-level analysis demonstrated that higher frequency of knee pain was significantly associated with greater varus or valgus deformity, increased limb-length discrepancy, and higher baseline Kellgren–Lawrence grades. These findings highlight the value of DL in elucidating the radiographic correlates of symptomatic OA progression and underscore the potential of AI tools to support large-cohort musculoskeletal research and personalized treatment planning (Kunze et al., 2023).

Beyond diagnostic support, AI has also proven valuable in patient selection for surgery. In total knee arthroplasty, deep learning models utilizing anterior–posterior, lateral, and patellar radiographs have been shown to accurately identify patients suitable for surgical intervention—a critical step in optimizing clinical outcomes (Liu et al., 2024). Additionally, AI-assisted three-dimensional (3D) preoperative planning has outperformed traditional two-dimensional (2D) templating methods in predicting prosthesis size, acetabular cup positioning, and lower-limb length restoration, further supporting its integration into orthopedic workflows (Wu et al., 2023a).

LLMs such as ChatGPT have also demonstrated utility in patient education and postoperative care, providing accurate, context-sensitive information to support at-home management following orthopedic procedures (Yapar et al., 2024). As imaging remains central to orthopedic diagnosis, deep learning models have consistently enhanced the diagnostic value of radiographs across multiple anatomical regions, particularly in the hip and extremities (Atalar et al., 2023; Erdaş, 2023).

Despite these advances, several limitations remain. LLMs may hallucinate or misrepresent content, fail to capture nuanced clinical contexts, or rely on outdated information—risks that are particularly concerning in high-stakes settings such as surgical planning or oncology (Pareek et al., 2024). Moreover, the “black-box” nature of many AI models continues to hinder interpretability, limiting their full integration into evidence-based clinical practice.

In summary, the integration of AI—through both deep learning and LLM-driven approaches—has substantially expanded the scope and sophistication of clinical decision support in orthopedics. Nonetheless, their implementation must be accompanied by rigorous validation, transparent methodology, and ongoing oversight to ensure safety, reliability, and practical utility across diverse clinical environments.

### 4.2 Medical education and patient communication

The emergence of large language models (LLMs), particularly ChatGPT, is reshaping both medical education and the communication dynamics between clinicians and patients. These tools offer significant potential in delivering structured content, simplifying complex medical terminology, and tailoring information to individual needs. Nevertheless, concerns persist regarding their reliability, factual accuracy, and ethical implications.

In medical education, LLMs are increasingly employed as supplementary tools that enhance access to clinical knowledge. ChatGPT, for example, has demonstrated the ability to generate case-based learning scenarios, summarize intricate guidelines, and provide real-time clarification of clinical concepts (Kayaalp et al., 2024). When used responsibly, these tools allow for individualized learning trajectories and help bridge knowledge gaps, particularly in resource-limited settings. A recent publication also highlighted ChatGPT’s value in overcoming linguistic barriers in scientific writing, offering critical support to nonnative English speakers (Kayaalp et al., 2024).

Despite these advantages, concerns remain regarding overreliance on AI in learning environments. Gültekin et al. (2025) reported that while ChatGPT-4o generated content-rich and comprehensive outputs for ACL-related patient education, the clarity and factual accuracy of its responses occasionally declined. This raises the risk of misinformation, particularly among learners lacking the clinical expertise or critical thinking skills to verify AI-generated content. Furthermore, excessive reliance on LLMs for exam preparation or assignments may reduce engagement with primary literature and weaken analytical reasoning skills.

To mitigate these risks, the integration of AI into medical training should be governed by well-defined pedagogical frameworks. Core competencies such as diagnostic reasoning, ethical judgment, and evidence-based practice must remain central to education. While LLMs can serve as valuable tools for rapid revision or clarification, they should not replace traditional pedagogical methods such as mentorship, problem-based learning, or supervised clinical training (Cascella et al., 2023).

Beyond professional education, LLMs have shown promise in improving patient communication. One of their most impactful applications lies in generating clear, personalized educational materials. By tailoring outputs to match a patient’s language proficiency and cultural context, LLMs can reduce misunderstandings and enhance treatment adherence. In a comparative study, both ChatGPT-4o and DeepSeek R1 generated comprehensive responses to orthopedic patient queries; however, differences emerged in clarity and readability, underscoring the trade-offs between informational depth and accessibility (Gültekin et al., 2025).

Similarly, ChatGPT’s performance in addressing patient-facing FAQs on hip arthroscopy was evaluated in a study in which responses were rated highly for clarity and informativeness, yet demonstrated low interrater reliability among orthopedic surgeons (Ayık et al., 2025). This underscores the importance of expert oversight in patient-facing applications.

As LLMs become more prevalent in shared decision-making, safeguards are essential. Outputs must be validated, up-to-date, and appropriately contextualized. Cascella et al. (2023) emphasized the need for robust monitoring protocols and regulatory frameworks, particularly when LLMs are integrated with electronic health records or patient portals.

Ethical considerations are also paramount. Issues such as algorithmic bias, data privacy, and the potential misuse of sensitive information necessitate governance mechanisms to ensure the ethical deployment of these technologies. As these tools become increasingly embedded in healthcare workflows, fostering digital literacy among both clinicians and patients will be critical to their safe and effective use.

In summary, LLMs such as ChatGPT and DeepSeek hold substantial potential to enhance both medical education and patient communication. However, their integration should complement—rather than replace—traditional instructional methods and clinical judgment. Responsible deployment must be anchored in transparency, validation, and ethical design to fully realize their value within the healthcare ecosystem.

### 4.3 Research assistance and scientific writing

The incorporation of large language models (LLMs) into biomedical research has transformed how clinicians and scientists approach academic writing, literature synthesis, and knowledge dissemination. ChatGPT and similar models are increasingly employed to support tasks such as manuscript drafting, citation verification, linguistic refinement, and the summarization of complex studies (Gültekin et al., 2025).

During the early stages of research, LLMs assist with identifying relevant literature, extracting core findings, and generating preliminary summaries. These capabilities are particularly useful for navigating dense and heterogeneous biomedical texts. With appropriately crafted prompts, LLMs can distill key insights from diverse sources and structure them into coherent narratives, improving efficiency while maintaining scholarly depth (Atik et al., 2024).

One of the most widely adopted applications of LLMs is in scientific writing. ChatGPT has been shown to substantially improve language clarity and grammatical precision, particularly benefiting researchers who are nonnative English speakers (Kayaalp et al., 2024). As a result, LLMs are increasingly regarded not merely as writing aids but as collaborative tools—copilots in scientific communication that streamline the transition from data to publication (Atik et al., 2024).

However, critical limitations remain. LLMs are prone to generating fabricated or inaccurate references, particularly when not guided by structured input. This issue underscores the necessity of human oversight at all stages of manuscript preparation. Without careful validation, there is a risk of academic misinformation and unintentional misconduct (Atik et al., 2024; Kayaalp et al., 2024).

Reproducibility and transparency also present challenges. Because LLMs typically do not cite specific source material unless explicitly prompted, tracing the origin of generated content can be difficult, raising concerns about verifiability and academic integrity (Zhou et al., 2024).

Furthermore, the growing reliance on AI for text generation raises ethical questions concerning authorship, plagiarism, and critical thinking. While LLMs can enhance fluency and assist with structuring content, they do not contribute original scientific ideas or conceptual frameworks. Therefore, they should not be considered coauthors or substitutes for scholarly reasoning (Leopold et al., 2023). Clear policies are needed to delineate the appropriate use of AI in research and to prevent the erosion of scientific rigor.

Looking ahead, the role of LLMs as research assistants is likely to expand. Emerging applications include automated screening for systematic reviews, formatting grant proposals, and generating lay summaries for broader public dissemination. To harness these capabilities responsibly, institutions and journals must establish transparent workflows and editorial standards for AI-assisted writing (Kayaalp et al., 2025).

In summary, LLMs such as ChatGPT and DeepSeek are now utilized across a broad spectrum of clinical and research tasks, offering versatile capabilities that span from surgical planning to academic writing. Table 1 provides a comparative overview of key clinical and research applications of LLMs in healthcare. Specific use cases of ChatGPT and DeepSeek in biomedical domains are summarized within Table 1 (Sagheb et al., 2021; Atik et al., 2024; Gültekin et al., 2025).

## 5. Large language models in biological sciences

### 5.1 Bioinformatics and omics

LLMs are driving transformative changes in bioinformatics and multiomics research by enabling automated interpretation of highly complex biological datasets. These models are capable of parsing and synthesizing vast volumes of genomic, proteomic, transcriptomic, and metabolomic data, providing actionable insights into molecular mechanisms and cellular functions. One of the most impactful applications of LLMs in this domain lies in their use for analyzing next-generation sequencing (NGS) data. LLMs facilitate the identification of disease-associated variants, the prediction of gene–gene interactions, and the detection of mutation patterns with high precision (Zrimec et al., 2020). By leveraging transfer learning and transformer-based architectures, these models can be fine-tuned on smaller, specialized datasets—an advantage particularly relevant to rare disease research, where data scarcity poses significant challenges (Jin et al., 2024).

Moreover, the integration of LLMs with convolutional neural networks (CNNs) has enabled the automation of advanced bioinformatics tasks such as transcript quantification, variant-effect prediction, and chromatin-structure annotation (Jin et al., 2024). This fusion of NLP and deep learning architectures has enhanced reproducibility and feature extraction from noisy biological signals, a limitation often encountered in omics analyses.

Unsupervised machine learning techniques also offer powerful tools for analyzing high-dimensional biological and clinical datasets. Methods such as k-means clustering, hierarchical clustering, principal component analysis (PCA), and factor analysis enable researchers to uncover latent patterns and simplify complex data structures. These tools are particularly valuable when labeled datasets are unavailable, facilitating hypothesis generation and subgroup identification in exploratory analyses. By integrating unsupervised learning into health sciences research, investigators can identify novel risk factors, improve disease stratification, and support the development of personalized treatment approaches (Eckhardt et al., 2023).

Generative models, including generative adversarial networks (GANs) and variational autoencoders (VAEs), further contribute by synthesizing biologically plausible datasets. These models enhance training pipelines through data augmentation while preserving patient privacy—an increasingly important consideration in translational research (Jin et al., 2024). However, despite their potential for integrating multiomics data, such generative models require extensive clinical validation to ensure robustness against dataset shifts, batch effects, and population heterogeneity (Kaczmarczyk et al., 2024).

Another emerging capability of LLMs is natural language querying of large-scale biological databases. Domain-specific tuning enables these models to extract contextually relevant summaries and generate hypotheses from unstructured biomedical literature, thereby bridging the interpretive gap between high-throughput data and biological insight (Jin et al., 2024).

In functional genomics, LLMs are beginning to support advanced tasks such as gene function annotation, pathway inference, and regulatory network modeling. When coupled with structured ontological frameworks such as the gene ontology (GO) or KEGG, they offer promising avenues for hybrid reasoning that integrates statistical learning with symbolic representations (Oeding et al., 2023a).

Despite these advancements, several challenges continue to limit broader adoption in clinical omics. These include the high dimensionality and heterogeneity of biological data, the risk of model hallucination, and persistent concerns regarding explainability. Nevertheless, progress in model transparency, the incorporation of biomedical ontologies, and the development of interpretable hybrid architectures is gradually addressing these issues.

As LLMs continue to evolve, their integration into multiomics research and personalized medicine is expected to expand. These tools are poised to play a central role in the next generation of biomedical discovery, systems biology, and translational research.

### 5.2. Synthetic biology and laboratory automation

The convergence of artificial intelligence with synthetic biology and laboratory automation is accelerating experimental workflows while enhancing precision, scalability, and reproducibility. Among the most impactful applications is the use of LLMs to generate experimental protocols, automate bioinformatics analyses, and streamline high-throughput experimental research.

LLMs have demonstrated the ability to produce structured laboratory protocols and documentation from unstructured prompts, effectively bridging the gap between experimental design and execution. For example, AI systems have been applied to automatically generate structured diagnostic reports—such as identifying meniscal tears on MRI—illustrating their practical relevance across both clinical and research settings (Güngör et al., 2025).

In synthetic biology, LLMs also support the design–build–test–learn cycle by generating code and documentation for experimental procedures. Pretrained models can translate human-readable prompts into executable scripts, aiding in the automation of complex workflows involving genetic circuit design, pathway engineering, and CRISPR screening. This capability is particularly valuable in high-throughput experimental platforms, where reproducibility and standardization are critical to success.

Laboratory automation has similarly benefitted from AI integration. Robotic systems, augmented by LLM-guided optimization algorithms, are now capable of conducting routine experimental tasks with minimal human intervention. These systems leverage closed-loop feedback from real-time data streams, enabling continuous performance improvement. In parallel, tools such as AlphaFold, developed by DeepMind, have revolutionized protein structure prediction, providing atomic-level accuracy that informs hypothesis generation and protein engineering workflows in synthetic biology (Jumper et al., 2021).

However, deploying these technologies requires robust bioinformatics knowledge, model validation procedures, and adherence to regulatory frameworks. Errors in AI-generated protocols or outputs—particularly in automated laboratory settings—may propagate undetected if not cross-validated with experimental controls. Therefore, responsible deployment necessitates version-controlled environments, benchmarking against curated datasets, and consistent human oversight to maintain scientific integrity.

Taken together, the synergistic application of LLMs, robotic laboratory platforms, and predictive modeling tools holds immense promise for synthetic biology. These technologies are not only accelerating discovery and reducing manual errors but also enabling new paradigms of reproducible, data-driven experimentation that are shaping the future of molecular biology and biotechnology.

## 6. The perils and limitations of large language models in healthcare

While LLMs such as ChatGPT and DeepSeek have introduced substantial advancements in clinical workflows, education, and biomedical research, their deployment also entails inherent limitations and risks, particularly when used without expert oversight. One of the most pressing concerns is the phenomenon of hallucination, wherein models generate fluent but inaccurate or fabricated information. In clinical contexts, such errors can undermine patient safety, lead to inappropriate treatment decisions, and erode trust in AI-supported systems (Jeyaraman et al., 2023; Gültekin et al., 2025).

These hallucinations are rooted in the probabilistic architecture of LLMs, which are trained to produce plausible sequences of words rather than to verify factual accuracy (Dave et al., 2023). Studies evaluating ChatGPT’s performance in clinical scenarios have identified frequent inclusion of outdated or oversimplified content, some of which diverged from current medical guidelines (Kaarre et al., 2023; Gültekin et al., 2025). The risk of misinformation is particularly pronounced in niche specialties or when addressing queries that depend on the most recent clinical evidence (Cascella et al., 2023). LLMs also face critical limitations in clinical reasoning and context integration. Despite their ability to process extensive medical literature, these models lack genuine situational awareness and struggle to synthesize multimodal data, including laboratory values, imaging, and longitudinal patient histories. As a result, they often generate superficial assessments that may overlook clinically relevant nuances (Cheng et al., 2023; Huang et al., 2024). For example, in a study evaluating ChatGPT’s ability to interpret radiographic findings in knee osteoarthritis, the model was found to underestimate disease severity and failed to provide clinically actionable insights (Zhu et al., 2025).

Similarly, machine learning models developed for predicting revision risk after hip arthroscopy using national registry data showed limited utility, highlighting the challenges of generalizability and clinical applicability (Martin et al., 2023).

Another major challenge pertains to ethical and social risks. LLMs trained on large-scale textual data may inadvertently learn and reproduce implicit biases related to race, sex, or socioeconomic status. Without effective bias mitigation strategies, these systems could propagate inequities in diagnosis or treatment recommendations (Wang et al., 2024). The growing accessibility of ChatGPT for health-related inquiries has also raised concerns about patients bypassing clinical consultations, potentially leading to unsafe self-management practices (Will ChatGPT transform healthcare?, 2023).

Data privacy and security present additional concerns. LLMs typically require access to large corpora, and if personal health data are not sufficiently anonymized, privacy breaches may occur. Furthermore, the integration of LLMs into clinical platforms—such as electronic health record systems—may introduce cybersecurity vulnerabilities if safeguards are not rigorously enforced (Morita et al., 2023).

In academic contexts, LLMs have improved access to scientific writing, particularly for nonnative English speakers. However, these same tools raise concerns regarding reference fabrication, plagiarism, and the dilution of academic authorship standards (Lund et al., 2023). These developments challenge traditional models of scholarly integrity and necessitate clear policy frameworks (Jeyaraman et al., 2023). Over time, scientific communities will inevitably need to adopt alternative approaches to chatbot integration in order to mitigate these adverse effects (Dahmen et al., 2023).

To address these limitations, multilayered safeguards are essential. These include human-in-the-loop (HITL) systems, regulatory oversight, explainability mechanisms, and clinician education on the appropriate use of AI. Ongoing auditing and model transparency are crucial to maintaining trust and accountability (Kelly et al., 2019; Kayaalp et al., 2025). To support safe and responsible use, various technical and procedural safeguards have been proposed. For instance, an AI-based computer-aided diagnostic system significantly improved both interrater agreement and diagnostic accuracy among board-certified orthopedic surgeons evaluating radiographic features of knee osteoarthritis (Smolle et al., 2023).

Several limitations of LLMs in healthcare have been identified in recent literature. One of the most critical issues is hallucination—where the model produces fluent but factually incorrect content—which poses significant safety risks in clinical applications (Lund et al., 2023; Smolle et al., 2023). Another key concern is user overreliance, wherein patients or clinicians may accept AI-generated answers without appropriate validation, potentially leading to harmful decisions (Jeyaraman et al., 2023).

Additional limitations include the opacity of model outputs, the lack of real-time clinical context integration, outdated training data, and the inability to handle nuanced or multimodal information. Moreover, ethical challenges such as algorithmic bias, unequal access, and data privacy vulnerabilities remain unresolved (Cascella et al., 2023; Kayaalp et al., 2025).

To mitigate these issues, several strategies have been proposed, including human-in-the-loop (HITL) oversight, domain-specific fine-tuning, integration with retrieval-augmented generation (RAG) systems, robust postdeployment auditing, and clinician education on responsible AI usage. These layered safeguards aim to improve accuracy, transparency, and ultimately trust in AI-supported medical workflows (Kelly et al., 2019; Dahmen et al., 2023; Morita et al., 2023).

With such measures in place, LLMs may serve as effective adjuncts to clinical care rather than as unreliable decision-makers. Their integration into healthcare will require a careful balance between innovation and responsibility. Used wisely, they can enhance efficiency and improve access to knowledge; used indiscriminately, they risk compromising patient safety, equity, and the foundational principles of medical ethics.

## 7. Deep learning perspective and clinical translation

Artificial intelligence and deep learning technologies are rapidly reshaping clinical practice and healthcare delivery. A key recent advancement is explainable artificial intelligence (XAI), which aims to clarify the internal mechanisms of deep learning models and address the long-standing challenge of algorithmic opacity. By increasing transparency, XAI enhances trust among clinicians and supports broader adoption of AI-assisted decision-making (Feierabend et al., 2025).

In clinical specialties, explainability is essential for model acceptance. Clinicians must be able to trace model predictions back to specific features, such as radiographic findings or clinical notes. For instance, deep learning models have demonstrated strong performance in detecting meniscal tears on MRI, yet the lack of interpretability limits their clinical integration (Güngör et al., 2025).

To overcome this, hybrid systems incorporating human oversight—commonly referred to as human-in-the-loop (HITL) frameworks—have been proposed. These systems enable clinicians to validate AI-generated outputs in real time and override recommendations when necessary, thereby improving safety and aligning decisions with established clinical guidelines.

The most frequent applications of AI in healthcare have been in diagnostic imaging and disease classification. In oncology, pathology, and radiology, AI has improved diagnostic accuracy and reduced time to diagnosis (Han et al., 2025). In orthopedic surgery, applications include preoperative templating, intraoperative navigation, and postoperative rehabilitation, all of which have improved procedural precision and workflow efficiency.

For example, in complex proximal humerus fractures, AI-based virtual reduction models have enabled faster planning and higher-quality anatomical alignment (Jeon et al., 2024). Similarly, AI tools used in total hip arthroplasty have shown improved accuracy in femoral stem placement compared to traditional planning methods (Mozafari et al., 2024). In patients with developmental dysplasia of the hip, AI-assisted three-dimensional preoperative planning significantly outperformed traditional two-dimensional templating in prosthesis size prediction and lower-limb length restoration, achieving a higher proportion of acetabular cups placed within established safety zones (Wu et al., 2023b; Przadka et al., 2025). A growing number of studies across various orthopedic subspecialties continue to report positive clinical outcomes associated with AI-based applications (Han et al., 2025).

Despite these achievements, real-world clinical deployment of deep learning models remains limited. A key barrier to broader adoption is the disconnect between clinical and technical expertise. Many orthopedic surgeons lack the computational training required to lead or collaborate effectively on deep learning initiatives (Oeding et al., 2023b). Supervised learning remains the most widely used machine learning approach in medical research, supporting classification and prediction tasks with known outcomes. Despite its clinical relevance, many healthcare professionals lack formal training in its application, underscoring the need for improved understanding of these methods to support their responsible use in orthopedic and broader healthcare research (Pruneski et al., 2023b). Models trained on single-institution datasets often underperform when applied to more diverse populations because of variability in imaging protocols and documentation practices. Additionally, algorithmic drift—where model performance degrades over time because of evolving data inputs—poses long-term challenges (Sahiner et al., 2023).

Ethical, legal, and regulatory concerns must also be addressed. These include ensuring informed consent for AI-assisted care, mitigating algorithmic bias, maintaining patient privacy, and enabling secure data sharing for multicenter research collaborations (Bozzo et al., 2024).

To foster adoption, AI models must prioritize transparency and clinician interpretability. Validation frameworks such as TRIPOD-AI and DECIDE-AI offer structured approaches to assess model readiness for clinical use. Their widespread implementation can help bridge the gap between computational promise and practical utility.

To better contextualize the real-world integration of LLMs and AI tools, Table 2 summarizes the clinical specialties in which LLMs such as ChatGPT have demonstrated tangible benefits, supported by domain-specific studies (Cascella et al., 2023; Zarei et al., 2024; Kayaalp et al., 2025).

In summary, deep learning has the potential to transform clinical medicine, but its success will depend on transparent design, rigorous evaluation, and sustained collaboration among developers, clinicians, and regulators.

## 8. Future directions of large language models in healthcare

The future of large language models in healthcare is marked by expanding integration into clinical decision-making, education, research, and global health delivery. One promising direction involves the development of multimodal AI systems that integrate LLMs with data from imaging, laboratory tests, and electronic health records. Such integration could yield more comprehensive and contextually aware clinical insights (Cascella et al., 2023).

For instance, AI-enhanced platforms used in preoperative planning have demonstrated improvements in implant selection and leg-length restoration for total hip arthroplasty (Wu et al., 2023a). Future models may integrate genomic profiles, social determinants of health, and real-time clinical data to advance precision medicine strategies (Dave et al., 2023; Wang et al., 2023).

Widespread adoption will require regulatory structures that ensure safety, accountability, and transparency. These include performance validation, bias detection, data security standards, and mechanisms for clinical oversight. Continuous monitoring and retraining will be necessary to maintain model relevance and accuracy (Choudhury and Chaudhry, 2024).

Ethical considerations remain essential. Transparent and interpretable models are critical to fostering clinician trust and ensuring appropriate patient engagement. This is particularly important in sensitive areas such as end-of-life care and mental health, where AI tools should complement rather than replace human judgment (Jeyaraman et al., 2023; Atik et al., 2024; Kayaalp et al., 2024).

In education, LLMs can be tailored to provide interactive case simulations, summarize complex topics, and deliver personalized learning modules. These tools can supplement medical curricula and support professional development; however, educators must ensure that foundational competencies such as critical thinking, ethics, and evidence-based reasoning are preserved (Clusmann et al., 2023).

LLMs may also enable more sophisticated applications in precision medicine. These models can assist in treatment planning by integrating multiomics data and identifying risk profiles in fields such as oncology and orthopedics (Gültekin et al.,, 2025). In surgical applications, AI-assisted robotics and patient-specific implants are already showing promise in early clinical studies (Naik et al., 2022; Zhou et al., 2025).

In low-resource or underserved regions, ChatGPT-powered telemedicine tools may help expand access to triage, diagnosis, and health education services. Translation features and culturally adaptive communication could reduce language and literacy barriers. However, digital access and infrastructure disparities must be addressed to ensure equitable impact (Wu et al., 2023b).

Ultimately, the future of LLMs in healthcare will depend on balancing innovation with responsibility. Their development and deployment must be guided by ethical governance, human-centered design, and rigorous clinical validation to ensure that these technologies serve all patients safely and effectively.

## 9. Conclusion

Artificial intelligence is increasingly embedded in healthcare through deep learning and large language models, offering novel capabilities in diagnosis, decision-making, and clinical communication. These tools have demonstrated value in interpreting complex datasets, supporting surgical planning, and improving access to scientific knowledge (Atalar et al., 2023; Erdaş, 2023).

In patient care, AI assists clinicians in preoperative counseling, helping patients understand indications, outcomes, and procedural risks (Kaarre et al., 2023; Yüce et al., 2024; Oeding et al., 2024; Gültekin et al., 2025). In research and publishing, LLMs support nonnative English speakers by improving manuscript clarity, enhancing citation accuracy, and streamlining literature synthesis (Kayaalp et al., 2024).

However, these benefits must be weighed against well-documented risks. Hallucinations, outdated information, and inaccurate recommendations remain persistent concerns, particularly in safety-critical environments (Jeyaraman et al., 2023; Kaarre et al., 2023; Martin et al., 2023; Gültekin et al., 2025). Overreliance on unverified outputs could misguide clinical decisions or compromise scientific rigor.

To address these risks, human-in-the-loop (HITL) frameworks offer a balanced approach by combining AI’s efficiency with expert oversight (Martin et al., 2023; Kayaalp et al., 2024). This model ensures accountability and maintains professional standards while benefiting from technological innovation.

As AI systems continue to evolve, their future role in healthcare must be shaped by a commitment to safety, inclusivity, and transparency. Properly developed and responsibly integrated, these technologies hold great promise for advancing healthcare quality, equity, and efficiency on a global scale.

## Figures and Tables

**Table 1:** Clinical and research use cases of large language models in healthcare.

Domain	Use case	Examples/applications
**Clinical Decision Support**	Symptom checking, differential diagnosis, and surgical planning	ChatGPT-assisted diagnostic suggestions, DeepSeek triage modeling
**Patient Communication**	Simplified health education, multilingual discharge summaries	ChatGPT-generated post-operative instructions, ACL education modules
**Medical Education**	Adaptive learning, case-based simulation, guideline summarization	Real-time anatomy/pathology explanations, USMLE prep
**Telemedicine**	Remote triage, language translation, clinical documentation generation	ChatGPT for rural or low-resource patient consultations
**Radiology/Imaging**	Automated fracture detection, alignment evaluation, severity grading	Detection of radius/ulna fractures, knee osteoarthritis interpretation
**Genomics/Omics**	Variant calling, pathway analysis, hypothesis generation	NLP-enhanced gene annotation, transcriptomics pattern recognition
**Manuscript Writing**	Drafting, summarization, citation checking, linguistic refinement	Abstract generation, grammar correction, citation formatting
**Systematic Reviews**	Automated literature screening, evidence extraction	LLM-guided PRISMA filtering and PICO extraction
**Precision Medicine**	Integration of clinical, imaging, and omics data for treatment planning	Personalized oncology or orthopedic risk profiling
**Protocol Generation**	Experimental workflows, laboratories automation scripting	Design-build-test-learn automation in synthetic biology laboratories

Sources: Adapted from Sagheb et al., 2021; Atik et al., 2024; Gültekin et al., 2025.

**Table 2:** Clinical specialties with the strongest evidence for artificial intelligence and large language model integration.

Specialty	Key AI applications	Representative evidence / studies
**Radiology**	Image classification, anomaly detection, and report generation	Deep learning for fracture detection [17]; FAI diagnosis [2]
**Orthopedics**	Surgical planning, alignment assessment, fracture identification, patient education	ACL revision risk prediction [28]; PTS measurement [65]; Meniscal tear detection [20]
**Oncology**	Pathology slide interpretation, cancer subtype classification, genomics-based decision support	Diagnostic accuracy in histopathology [6]; Genomic integration [72]
**Cardiology**	ECG interpretation, heart failure prediction, imaging analytics	AI in echocardiography; arrhythmia detection (literature-based)
**Neurology**	Stroke detection, brain MRI segmentation, cognitive disorder screening	Deep learning in neuroimaging (generalizable studies exist)
**Dermatology**	Skin lesion classification, melanoma detection	CNN-based skin image classifiers (FDA-approved tools)
**Primary Care/Internal Medicine**	Symptom triage, differential diagnosis, chatbot-assisted patient interaction	LLM-based chat tools (e.g., ChatGPT vs physician triage studies)
**Medical Education/Scientific Writing**	Adaptive learning, language simplification, abstract drafting	ChatGPT for USMLE performance [38]; Language refinement [34]

Sources: (Morita et al., 2023; Jeyaraman et al., 2023; Cascella et al., 2023; Zarei et al., 2024; Kayaalp et al., 2025)

## Footnotes

1 Densen, P. (2011). Challenges and opportunities facing medical educationTransactions of the American Clinical and Climatological Association, 122, 48-58 [online]. Website https://www.ncbi.nlm.nih.gov/pubmed/21686208 [accessed 17 August 2025]

2 Lowe, D. (2025). An Evaluation of “Deep Research” Performance [online]. Website https://www.science.org/content/blog-post/evaluation-deep-research-performance [accessed 08 August 2025]
